# Bluetongue Virus Serotype 8 Reemergence in Germany, 2007 and 2008

**DOI:** 10.3201/eid1409.080417

**Published:** 2008-09

**Authors:** Bernd Hoffmann, Michael Saßerath, Sabine Thalheim, Claudia Bunzenthal, Günter Strebelow, Martin Beer

**Affiliations:** Friedrich-Loeffler-Institut, Greifswald-Insel Riems, Germany (B. Hoffmann, G. Strebelow, M. Beer); State Veterinary Institute Krefeld, Krefeld, Germany (M. Saßerath, C. Bunzenthal); Landeslabor Brandenburg, Frankfurt (Oder), Germany (S. Thalheim)

**Keywords:** Bluetongue virus, BTV-8, re-occurrence, dispatch

## Abstract

Reemerging bluetongue virus serotype 8 (BTV-8) in Germany was detected first in May 2007 in a sentinel cow and in February 2008 in an export heifer. Reemergence was confirmed by retesting the samples, experimental inoculation, fingerprinting analysis, and virus isolation. Overwintering of BTV-8 and continuous low-level infections are assumed.

Bluetongue virus (BTV) is a double-stranded RNA virus of the genus *Orbivirus.* BTV is transmitted to its hosts by the bite of *Culicoides* spp. midges. It causes a noncontagious, arthropod-borne disease of domestic and wild ruminants and camelids (*1*); disease can be serious, particularly in sheep (*2*–*4*). BTV had never been reported in any European country north of the Alps until August 2006, when outbreaks of BTV serotype 8 (BTV-8) were almost simultaneously discovered in the Netherlands, Belgium, Germany, and France (*5*–*7*). In 2006, a total of 893 cases were detected in Germany, but the source of initial virus introduction remains unknown. Subsequently, BTV-8 overwintered in the region, spread over most of the country, and led to almost 21,000 new cases in 2007 in Germany. BTV-8 infections spread to additional European countries, e.g., the United Kingdom, Switzerland, the Czech Republic, Denmark, and Spain (*8*–*11*).

To detect reemerging BTV-8 in Germany, a sentinel animal program was started in March 2007. Each month, the program tested 150 BTV-naive cattle per 2,025 km². Screening was performed by ≈30 regional laboratories located in the 16 federal states of Germany. Diagnostic testing was performed with commercially available BTV-blocking-ELISAs (Pourquier ELISA Bluetongue Serum, Institut Pourquier, Montpellier, France; Bluetongue Virus Antibody Test Kit, VMRD, Pullman, WA, USA: ID Screen Bluetongue Competition ELISA kit, ID Vet, Montpellier, France; INGEZIM BTV, INGENASA, Madrid, Spain) and validated BTV real-time PCRs (*12*; B. Hoffmann et al., unpub. data). All positive results were confirmed by the National Reference Laboratory for BTV at the Friedrich-Loeffler-Institut, Insel Riems.

## The Cases

On May 3, 2007, the first sentinel animal in the program (4-year-old Holstein Friesian cow, North-Rhine Westphalia, Germany) tested positive for BTV antibodies and genomes in a regional laboratory (Krefeld, Germany). This animal had tested negative for BTV on March 3 and April 3. After the positive test result, the original samples as well as new samples were transferred to the reference laboratory for further investigation. The sample collected on May 3 and all further samples of this animal tested positive by BTV ELISA as well as by BTV real-time reverse transcription–PCR (RT-PCR). According to Deutscher Wetterdienst, available from www.dwd.de, the daily temperatures in the region of North-Rhine Westphalia during this period ranged from minimums of 13.5°C to –1.3°C to maximums of 9.5°C to 28.1°C. A period with daily maximum temperatures >22°C from April 12 to April 16 suggests increased likelihood for activity of *Culicoides*. Similar prognoses for the reemergence of BTV-8 were published by Gloster et al. (*13*).

Samples taken on May 3 had positive ELISA titers of 8–16, and the cycle threshold (Ct) values of the real-time RT-PCR analysis were 25.6–27.9 (Figure, panel **A**). Therefore, new BTV-infection of a BTV-naive animal was suggested. To confirm the presence of infectious BTV, fresh EDTA-blood samples from the positive animal were experimentally injected into 2 bovids (40 mL intravenously and 10 mL subcutaneously). To mimic natural exposure by the bite of a midge, only 20 µL was injected subcutaneously into a 3rd bovid (Table). Both animals inoculated with 50 mL had positive real-time PCRs at day 2 postinoculation and reached Ct values of 22.9 and 24.3 by day 13. In contrast, the animal that had received the smaller volume remained negative for BTV antibodies and genomes. In addition, 1 of the animals that had received the larger volume showed late seroconversion according to BTV-blocking ELISA at day 30 (Table). Furthermore, the virus was typed as BTV-8 by using the BTV-8–specific primers BT8/101/F (*14*) and BT8/765/R (5′-CCACTGTCTATGTG CGTAA-3′, kindly provided by P. Mertens, Institute for Animal Health, Pirbright, UK) to amplify a 1,993-bp fragment of the VP2 gene by conventional RT-PCR (Figure, panel **B**). Testing of 80 contact animals from the same herd showed 38 antibody-positive cattle but no additional RT-PCR–positive case.

**Figure:**
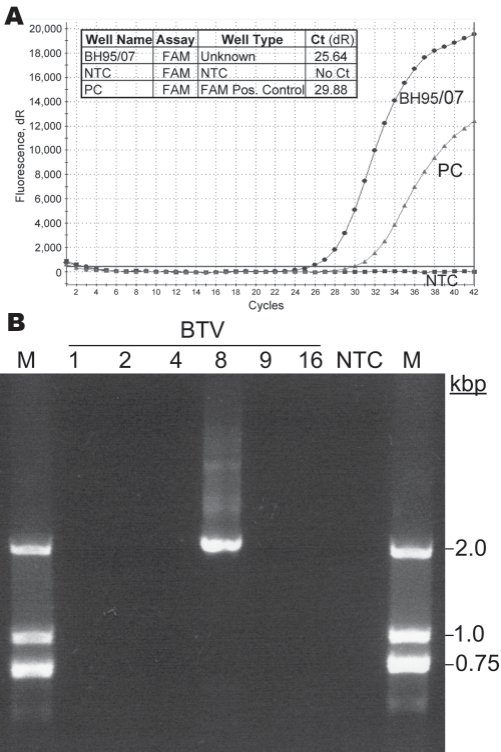
A) Bluetongue virus (BTV) serotype 8 genome in a field sample collected May 3, 2007 (BH95/07), detected by using real-time reverse transcription–PCR with a 6-carboxy fluorescein-labeled probe (FAM). The magnitudes of the fluorescence signals per PCR cycle are shown for the sample (Unknown), the no-template control (NTC), and the positive control (PC). Ct, cycle threshold. B) Confirmation of serotype 8 for the isolated virus by serotype-specific PCR and agarose gel analysis. Additionally tested serotypes (BTV 1, 2, 4, 9, 16) were negative. A DNA ladder was used as marker (M).

**Table:** Results of rRT–PCR and bluetongue virus competition ELISA of samples from experimentally inoculated animals*

Day postinoculation	Animal no., dose							
	415, 40 mL IV and 10 mL SC			418, 40 mL IV and 10 mL SC			419, 20 μL SC	
	rRT-PCR, Ct	ELISA		rRT-PCR, Ct	ELISA		rRT-PCR, Ct	ELISA
2	>42.0	–		>42.0	–		>42.0	–
4	37.7	–		37.2	–		>42.0	–
6	32.1	–		28.7	–		>42.0	–
9	30.9	–		26.6	–		>42.0	–
13	24.3	–		22.9	?		>42.0	–
16	24.3	–		22.8	+		>42.0	–
21	26.7	–		24.5	+		>42.0	–
30	27.0	+		27.2	+		>42.0	–

*rRT-PCR, real-time reverse–transcription PCR; IV, intravenous; SC, subcutaneous; Ct, cycle threshold (Ct values >42 were defined as negative); –, negative; +, positive; ?, in doubt.

In February 2008, a 2-year-old Holstein Friesian heifer, destined for export from Schleswig-Holstein, tested positive for BTV antibodies and genomes during quarantine. Testing was done by a regional laboratory (Frankfurt Oder, Germany). Whereas a first blood sample collected on January 22, 2008, was negative according to real-time RT-PCR; a subsequent sample taken on February 15 was positive by real-time RT-PCR and had a Ct value of 25.13. Both test results were confirmed by the German National Reference Laboratory for BTV. For the positive EDTA blood sample, a Ct value of 25.1 and an ELISA inhibition value of 85.7% (positive >50%) were ascertained. Furthermore, fingerprinting analysis (StockMarks Cattle Genotyping Kit, Applied Biosystems, Foster City, CA, USA) confirmed that both the negative and the positive samples were from the same animal (data not shown). The meteorologic data from January 20 to February 15 showed unsuitable weather conditions for BTV replication in outdoor midges at the time of BTV transmission; daily minimum temperatures were 7.2°C to –6.3°C, and daily maximum temperatures 3.4°C–13.4°C. Finally, BTV was isolated after inoculation of Vero cell cultures with the real-time PCR-positive blood samples.

## Conclusions

According to the dates of sampling and subsequent seroconversion, genome detection, and Ct values, 1 animal became infected during April 1–25, 2007, and the other became infected during January 20–February 12, 2008. According to meteorologic data, infection between April 15 and 25 is likely, whereas the suggested infection time in 2008 was not associated with weather suitable for BTV transmission. Therefore, we have drawn several conclusions. First, BTV-8 reemerged in Europe in 2007 and 2008 unexpectedly early. This timing makes it unlikely that a real vector-free period exists in northwest Europe. Second, the detection of both cases was fortuitous. Because of the low number of sentinel animals in 2007, or double-tested cattle destined for export in January and February 2008, substantially more new infections may have occurred in winter 2007 and early spring 2008. Therefore, overwintering of BTV-8 appears to be efficient, and under the climatic conditions of winter and spring 2007 and 2008 in Germany, continuous infections occurring at a low level must be assumed. However, the detailed mechanisms of overwintering remain unclear. In addition, the meterologic data connected to the early case in 2008 suggest indoor survival of infected midges. Third, sentinel animals proved to be useful for the early detection of recirculating virus. However, establishing and conducting an efficient sentinel program are complex and expensive endeavors, and, as shown in 2008, positive cases might be detected earlier by chance.

On the basis of our experience, we recommend that all samples that could represent new infections in a given region or season should be confirmed by a reference laboratory that as a minimum standard confirms identity of the origin of all samples. This step would avoid misleading interpretations due to, for example, a mix-up of samples or animals. Also, depending on future climate in the BTV-8–infected regions in 2008, the first BTV-8 case could indicate the need for early protection, especially of sheep and goats, by BTV-8–specific vaccination.
